# Understanding Sickle Cell Disease Endothelial Pathology Through the Lens of Liver Sinusoidal Endothelial Cells

**DOI:** 10.3390/cells15090846

**Published:** 2026-05-05

**Authors:** Tirthadipa Pradhan-Sundd, Brian Branchford, Joan Beckman

**Affiliations:** 1 Thrombosis and Hemostasis Research Program, Versiti Blood Research Institute, Milwaukee, WI 53226, USA; 2 Division of Hematology, Oncology, and Transplantation, Department of Medicine, University of Minnesota, Minneapolis, MN 55455, USA; beckm092@umn.edu

**Keywords:** sickle cell disease, endothelial dysfunction, liver sinusoidal endothelial cells, hemoglobin, LSEC senescence

## Abstract

**Highlights:**

**What are the main findings?**

- Endothelial dysfunction is a hallmark of sickle cell disease (SCD), which is associated with both acute and chronic crises in patients.
- Whereas lung or kidney endothelial beds are widely studied in SCD, liver sinusoidal endothelial cells are recently gaining attention for their previously unrecognized roles. This review discusses the function and regulation of LSECs in SCD.

**What is the implication of the main finding?**

- Understanding the molecular processes of LSEC-driven hemoglobin clearance and senescence may identify new biomarkers and regulators of acute and chronic liver damage in SCD.
- Modulations of LSECs scavenging activities may emerge as a potential therapeutic option for liver damage associated with SCD.

**Abstract:**

Endothelial cell activation is one of the major pathophysiological aspects of sickle cell disease (SCD). In this review, we discuss the interaction of endothelial cells of various tissue beds, highlighting the specific biomarkers linked to endothelial cell activation and damage, and elaborate on endothelial cells’ role in the development of acute and chronic organ damage in SCD using existing clinical and preclinical data. Finally, we focus on liver sinusoidal endothelial cells (LSECs) and their role in hemoglobin scavenging, sterile inflammation, and organ damage in SCD, as well as potential therapeutic strategies to reverse organ damage induced by LSEC dysfunction in SCD.

## 1. Introduction

Sickle cell disease (SCD) is an autosomal recessive genetic disorder that affects around 100,000 African Americans and more than 7 million people worldwide [1]. At the molecular level, SCD is defined by a sequence of events: a single point mutation in the β-globin chain of hemoglobin (Hb) results in the substitution of adenine to thymine, altering the hydrophilic amino acid glutamic acid with hydrophobic valine, which promotes the formation of β-globin chain interactions leading to polymerization of Hb and sickling of red blood cells (RBCs) [2]. Sickled RBCs undergo hemolysis at low oxygen concentration, releasing cell-free Hb, which functions as a damage-associated molecular pattern (DAMP) and activates cell adhesion molecules (CAMs) on the surface of the endothelium, causing endothelial damage [3,4].

Endothelial cell (EC) activation and damage are hallmarks of SCD pathophysiology. Endothelial damage is linked to many underlying complications of SCD, including acute chest syndrome, pulmonary hypertension, stroke, priapism, decreased renal function, and increased mortality [3]. Both invasive and noninvasive approaches for endothelial damage mitigation have demonstrated little success in SCD [4,5]. Thus, an improved understanding of the molecular mechanisms underlying endothelial activation-driven organ damage, as well as the identification of novel therapeutic interventions or biomarkers, is critical for reducing organ damage-associated mortality in SCD. In this review, we discuss endothelial damage-associated pathophysiology in SCD, with a specific focus on the liver sinusoidal endothelial cells (LSECs).

## 2. Activated Endothelial Cell Interactions in SCD

The activation of ECs is a key pathologic feature associated with SCD. RBC hemolysis, vascular damage, and activation of inflammatory molecules or platelets are the predominant causes of EC activation in SCD [4,6]. The ECs stay in a continuous state of activation in SCD and express a unique set of markers on their cell surface, including cellular adhesion molecules (CAMs) such as VCAM1, ICAM1, P-selectin, and E-selectin [7]. RBC-derived cell-free Hb interacts with these CAMs, promoting sterile inflammation and vaso-occlusion. Hb metabolites (ferric methe-hemoglobin) released in the plasma scavenge nitric oxide (NO), which causes oxidative stress and production of reactive oxygen species (ROS), further promoting EC activation and endothelial damage in SCD [6,8].

**Figure 1:**
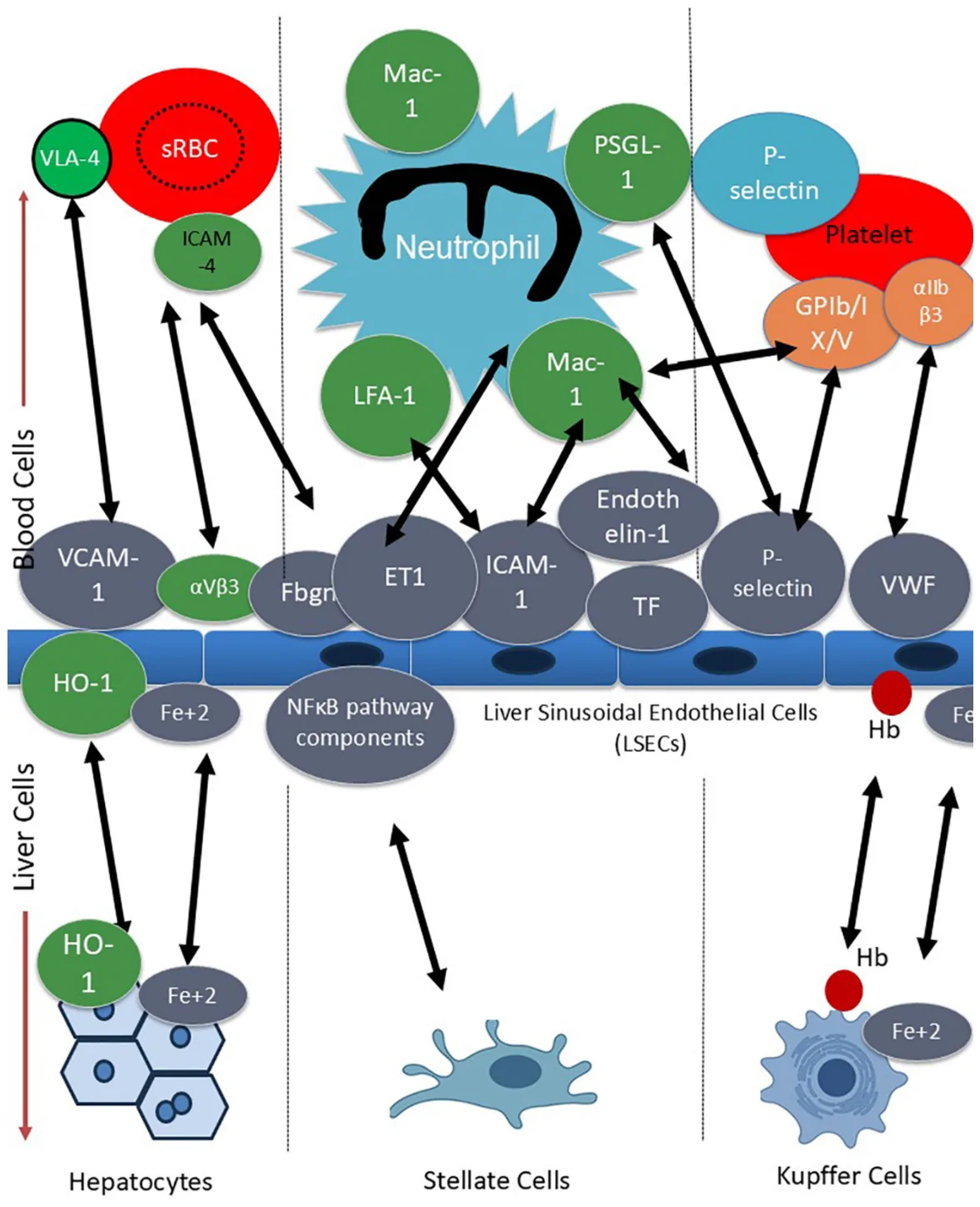
Activated endothelial cell interactions with other blood cell types in SCD. Schematic showing the interaction of activated endothelial cell markers with other blood (RBCs, neutrophils, and platelets) and liver cell types (hepatocytes, stellate cells, and Kupffer cells) reported in sickle cell disease. Abbreviations used for red blood cells (RBCs), heme oxygenase 1 (HO1), von Willebrand factor (VWF), intercellular adhesion molecule 1 (ICAM1), lymphocyte-associated antigen 1 (LFA1), very late antigen 4 (VLA4), endothelin 1 (ET1), p-selectin glycoprotein ligand 1 (PSGL1), tissue factor (TF), and glycoprotein 1bα. The arrow indicates reported interactions between different proteins. Image references: [3,7,19,29,30,31,32,33,34,35,36,37].

Activated ECs interact with various blood cell components in SCD, including RBCs, neutrophils, and platelets (*as illustrated in* *Figure 1*) [2,9,10,11]. RBC-EC interaction is a common phenotype linked to vaso-occlusive pain crisis in SCD patients [9]. Both static and flowing RBCs exhibit enhanced adherence to ECs, which positively correlates with numerous acute and chronic complications of SCD (such as acute chest syndrome, pulmonary hypertension, stroke, and kidney damage) [12]. RBC-EC interaction has also been connected to the release of microvesicles from the ECs and RBCs to the plasma of SCD patients [9,13]. Endothelial microvesicles contain tissue factor (TF) and phosphatidylserine and facilitate interactions between heme and ECs. Increased accumulation of endothelial microvesicles positively correlates with end-organ damage, exacerbated hemolysis, tissue hypoxia, pulmonary systolic pressure, and mortality in SCD [14,15].

Another common pathophysiology associated with endothelial damage in SCD is EC–neutrophil interactions [16]. Neutrophils aggregate to SCD endothelium in models of ischemia–reperfusion damage, sterile inflammation, and exacerbated vaso-occlusion [17]. Neutrophil–EC interactions are also common in bacterial infections [18]. At the molecular level, ligands present in the neutrophil (PSGL1 and ESL1) bind to endothelial receptors P- and E-selectin, forming neutrophil–EC aggregates [19]. Endothelin-1 (ET-1), an endothelium-derived vasoactive mediator, is increased in the plasma of SCD patients. Interactions of neutrophils with ET-1 cause activation of aggregated neutrophils [20]. Previous studies have established a link between SCD pain crisis and ET-1 neutrophil interaction. Mac1 (CD11b/CD18) present on the neutrophil surface promotes neutrophil–EC-RBC adhesion and vaso-occlusion in SCD [21].

Endothelial damage is also associated with increased EC–platelet interactions in SCD [22]. SCD patients’ plasma samples exhibit an increased number of circulating platelets. These activated platelets are inflammatory in nature and cause increased adhesion of neutrophils around them [23]. Platelet-EC adhesion is linked to the risk of stroke and vaso-occlusive pain crisis in SCD [15,24,25,26,27,28]. At the molecular level Akt pathway is shown to be activated in neutrophils and platelets, causing increased adhesion of platelet–EC–neutrophil adhesion in SCD [2]. NLRP3/BTK signaling was also found to be activated in platelets of SCD patients at baseline and post-acute crisis. Blocking members of these signaling pathways has been shown to alleviate inflammation, endothelial damage, and pain crises in SCD, suggesting their therapeutic potential [10,11,28]. A recent study has shown that enhanced EC adhesion to other blood cells activates pro-inflammatory reactions, pro-coagulant effects, and loss of endothelial barrier characteristics in SCD, which contributes to microvascular occlusion. Microvascular blockage causes hypoxia/ischemic physiology and changes in shear stress, modifying mechanical homeostasis within organs [25].

## 3. Endothelial Damage-Associated Biomarkers in SCD Patients

Endothelial damage in SCD patients has been a major topic of research interest for many decades, and endothelial damage-related biomarkers have emerged as a promising new therapeutic option in SCD patients. Mostly, endothelial damage-associated biomarkers include activated CAMs, increased release of microparticles and extracellular vesicles, coagulation and prothrombotic markers, intercellular cytokines, and NO scavengers, which are listed in Table 1.

A primary pathophysiological aspect of endothelial injury in SCD patients is the presence of circulatory endothelial cells (CECs) and endothelial progenitor cells (EPCs) [14,38]. SCD patients demonstrate elevated levels of CECs and EPCs in the blood plasma, indicative of vascular damage and activation of ECs [7,14]. In end-organ damage conditions such as stroke and renal failure, the CEC transcription profiles display distinct variations among SCD patients, suggestive of heterogeneity in these cells [39]. Elevated CECs are also linked to coagulation defects in SCD. TF is overexpressed on SCD CECs [33]. Like CECs, EPCs also show unique heterogeneity, aberrant transcriptional profiles, and the presence of angiogenesis-related genes in SCD patients [40]. Together, these studies suggest the structural–functional complexity of CECs and EPCs and their potential role as biomarkers for end-organ damage in SCD.

Beyond the shedding of CEC and endothelial microvesicles in the plasma, endothelial dysfunction in SCD also contributes to coagulopathy. Coagulation pathway biomarkers associated with endothelial damage in SCD include increased expression of TF [41], D-dimer [42], and fibrinogen [43] in the plasma, causing a prothrombotic, procoagulant phenotype in SCD patients. Among other coagulation-related proteins, SCD patients show an increase in complement C3a and C5a, a decrease in anticoagulant protein S and protein C, and an overall increase in fibrinolytic activity [44]. *Table 1 lists the endothelial damage-associated changes in coagulation proteins in SCD patients.*

**Table 1:** List of SCD-related activated endothelial cell markers and their presence in LSECs.

SCD Endothelial Marker Subgroups	Marker Name	References	Present in LSEC
Cell Membrane and Adhesion	ICAM1	[45]	+
	VCAM1	[46]	+
	PECAM1	[47]	+
	VE-Cadherin	[48]	+
	p-selectin	[49,50,51]	+
	e-selectin	[52]	+
	Lyve1	[53]	+
	CD31	[7]	+
	eNOS	[30]	+
	iNOS	[30,54]	+
	NO signaling molecules	[55]	+
Inflammatory	Endothelin1	[56,57,58]	+
	TNFα	[59,60]	+
	IL1	[60]	+
	IL6	[61]	+
	IL8	[62]	+
	MMPs	(preliminary data)	+
	MAC1	[21]	−
Coagulation	D dimer	[42]	+
	VWF	[19,63]	+
	Protein C	[64]	+
	Protein S	[65]	+
	APC	[66]	+
	Fibrinogen	[42,65,67]	+
	FVIII	[68]	+
	Tissue factor	[64,65]	+
	PAR1	[69]	+
	Thrombomodulin	[70]	+
	PAR complex	[66]	+
Other Biomarkers	CECs (undetected)	[7,71]	−
	microvesicles related	[7]	−

## 4. Organ-Specific Endothelial Damage in SCD

EC activation, and its downstream consequences, such as diminished nitric oxide activity and increased hypoxia, induce multiorgan damage in SCD [55]. Renal, lung, and brain endothelial dysfunction are prominent in SCD. Lung endothelial cell damage is a known factor for both chronic (pulmonary arterial hypertension [PAH], hypoxemia, and exercise intolerance) as well as acute (acute chest syndrome [ACS], pulmonary thromboembolism) lung damage seen in SCD [8,72]. Numerous studies have shown that lung endothelial damage-mediated exacerbations of vaso-occlusive pain crisis, ROS activation, and sterile inflammation promote PAH in SCD [3,8,73]. SCD mice exposed to lipopolysaccharide (LPS) mimic the ACS symptoms seen in SCD patients, exhibiting impairment of pulmonary endothelial barrier function [74], suggestive of sterile inflammation as one of the primary causes of lung endothelial damage in SCD. Likely due to free heme-related activation of MD2-toll-like receptor 4 (TLR4) activation in the endothelium [75]. In both SCD patients and preclinical mouse models, prior research has shown that endothelin-1 (ET-1) promotes ACS [20]. ACS is associated with increased markers of coagulation, including tissue factor, fibrin, and D-dimer [5]. SCD patients with ACS also demonstrated an increase in eNOS expression and enzymatic activity, suggesting that disruption of the NO signaling in acute lung endothelial damage in SCD [73]. Preclinical mouse research has also been useful in deciphering the molecular mechanism of lung endothelial damage, including dysregulation of tight junction protein ZO1 and endothelial membrane protein kazrin, causing endothelial membrane defects and loss of barrier function [3].

Endothelial damage-associated renal injury is common in SCD patients [56]. Studies done in both mice and humans have found that ET-1 is markedly elevated and causes renal damage in patients with SCD by inducing oxidative stress in SCD through its vasoconstrictor activity [56]. Activated CECs were also observed in SCD renal injury models [56]. Moreover, there is a positive correlation between increased endothelial microvesicle release and kidney vaso-occlusion in SCD [35]. SCD patients also exhibit endothelial damage-driven albuminuria, loss of kidney endothelial junctional proteins, deposition of complements in renal tissue, and elevated expression of EPCs [76,77].

Brain endothelial damage is associated with loss of the blood–brain barrier and increased permeability, which is associated with mortality in both children and adults with SCD [78]. Ischemic stroke is a potentially life-threatening complication in children with SCD, whereas adults more commonly suffer hemorrhagic stroke, though both are associated with endothelial damage-associated brain damage [3]. At the molecular level, brain endothelial damage shows increased expression of cellular adhesion proteins [78], nitric oxide dysfunction, endothelial shear stress, activation of mast cells [79], and increased ER stress.

While the pathophysiology of endothelial dysfunction in the lung, kidney, and brain has been studied extensively over the years, other organ-specific ECs are increasingly being recognized due to the rising life expectancy of SCD patients over the past decade and the associated increase in organ damage-related hospitalizations. Sickle-related liver disease is a frequent, but often under-recognized, complication of SCD [80,81,82,83]. Overall, 50–69% of SCD patients demonstrate evidence of liver involvement, such as elevated liver enzymes [81,82,83]. Acute hepatic sequestration occurs in 10% of individuals; however, in patients who reach 40+ years of age, cirrhosis and iron overload-induced damage are common and account for ~11% of SCD-related mortality [80]. However, the specific role of LSEC dysfunction in SCD remains understudied, leaving a significant gap in understanding how LSECs drive the progression of liver microvascular congestion to irreversible hepatopathy. Crucially, liver endothelial cells are not a homogeneous population; recent transcriptomic evidence has found that endothelial subtypes have unique functional trajectories. As the liver plays a prominent role in modulating cell-free hemoglobin clearance, liver sinusoidal endothelial cells (LSEC) likely contribute significantly to SCD pathogenesis [23,84,85,86,87,88]. Moreover, LSECs act as a primary site for the balanced synthesis and clearance of coagulation factors, suggesting that LSEC dysfunction may also contribute to sickle cell coagulopathy. Due to the identification of its many unknown roles in SCD, LSEC studies are gaining attention.

## 5. Liver Sinusoidal Endothelial Cells (LSECs) in SCD Disease Pathophysiology

LSECs are specialized ECs that exist at the interface between blood cells and hepatocytes [89]. LSECs not only create a barrier within the hepatic sinus but also perform crucial physiological tasks, including the regulation of hepatic vascular pressure, as well as exhibiting anti-inflammatory and anti-fibrotic properties [90]. LSECs constitute approximately 3% of the overall hepatic cell volume. Their distinct phenotypic characteristics, such as the lack of a basement membrane, the presence of endothelial fenestrae or pore-like structures, and the formation of a semipermeable barrier, have rendered them challenging to study for an extended period [91]. LSECs exhibit functional flexibility in addition to morphological differences when compared to other EC types in the body. They demonstrate exceptional scavenging capabilities [89]. One of their primary functions is the scavenging or endocytosis of proteins for destruction. LSECs possess various scavenger receptors, such as stabilins, mannose receptors, and lectins [92]. Noteworthy functions include the management of the hepatic milieu, stimulation of hepatic stellate cells (HSCs), and modulation of inflammation [91]. LSEC malfunction is linked to liver fibrosis, diverse hepatic metabolic abnormalities, alcoholic and non-alcoholic liver dysfunction, and the aging process [89]. As previously stated, LSECs have not yet been extensively examined in the context of SCD. Here, we summarize the effects of SCD-related hemolysis, sterile inflammation, and vaso-occlusion on LSECs, drawing from prior research on SCD patients and preclinical mouse models.

### 5.1. LSECs Do Not Exhibit Any Gross Structural Deformity in SCD Mice

**Figure 2:**
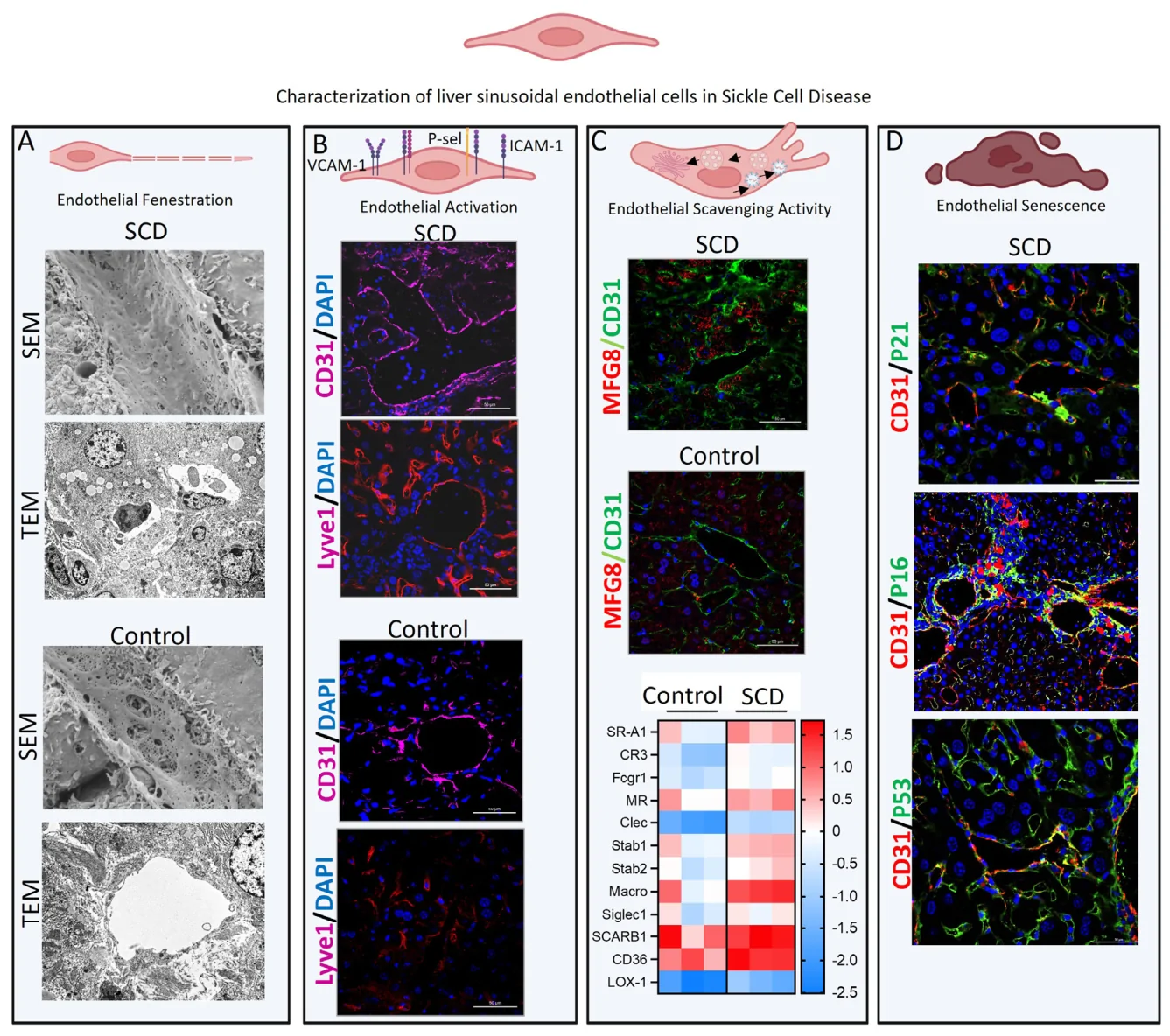
Characterization of liver sinusoidal endothelial cells in the Townes SCD mouse model. (**A**) Scanning electron microscopy (SEM) and transmission electron microscopy of control and SCD mouse livers demonstrate the endothelial fenestration, width of the liver sinusoids, and overall LSEC architecture, which appears comparable in both control and SCD mouse livers. (**B**) Representative IF images exhibiting activation of LSECs in SCD as compared to control mouse livers. CD31 (purple) and Lyve1 (red) are used as markers of LSECs, and cell nuclei are stained with DAPI (blue). (**C**) Representative IF images exhibiting the expression of autophagy marker MFG8 (red) in LSECs (marked with CD31, green) of SCD and control mouse liver. Cell nuclei are stained with DAPI (blue). Heatmap exhibiting the mRNA expression of the scavenger receptors present in the control and SCD mouse liver. Representative IF images exhibiting the localization of senescence markers P21, P16, and P53 (green) in LSECs (marked with CD31, red) of SCD and control mouse liver. Cell nuclei are stained with DAPI (blue). (**D**) Image references: [50,53,93,94,95,96,97]. Created in BioRender. Pradhan, T. (2026) https://BioRender.com/4n7879x.

While data on SCD patients’ LSECs are limited, there are a few reports of LSEC dysfunction in the preclinical SCD Townes mouse model. Unlike vascular damage and hepatic fibrosis seen in SCD, LSECs do not exhibit any gross morphological defect in SCD mice. As demonstrated in Figure 2A, transmission electron microscopy (TEM) and scanning electron microscopy (SEM) revealed a comparable number of fenestrae/field of view, and number of grouped pores/field of view in both SCD and littermate control mice. Interestingly, the sinusoidal diameter, as shown in Figure 2A, appeared slightly broader in SCD mice, potentially due to RBC sequestration and trapping inside the liver sinusoids. Together, these data suggest that LSECs do not have a significant structural deformity in SCD mice. It also suggests that RBC sequestration might be responsible for creating stress in the sinusoidal vessel wall, resulting in its increased diameter. Given that aberrations in vessel diameter can impair the endothelial barrier, future research focusing on the correct evaluation of LSECs’ barrier function may provide substantial progress toward understanding the effect of SCD pathophysiology on organ-specific endothelial dysfunction.

### 5.2. LSECs Are Activated in SCD Mice

We and others have shown that, similar to any other ECs of different vascular beds, LSECs of SCD mice show activation of endothelial markers, including VCAM1, ICAM1, P-selectin, E-selectin, Lyve1, CD31, and members of NO signaling, including iNOS and eNOS, at baseline (Figure 2B, Table 1) [50,95]. Moreover, SCD-induced hypoxia, increased inflammation associated with vaso-occlusion, or hemolysis can cause a further enhancement of these LSEC activation markers, which positively correlate with exacerbated liver damage [53,95]. Thus, LSEC activation could be one of the underlying causes of liver damage in SCD.

Among the endothelial markers, p-selectin activation is widely studied in SCD mice and patients [23,50,98,99,100]. Interestingly, SCD patients and mice exhibit increased expression of both endothelial- and platelet-derived p-selectin, which positively correlates with increased vaso-occlusion [49,98]. We have shown that blocking p-selectin in SCD mice caused LSEC dysfunction, senescence, and exacerbated liver damage [50]. As the p-selectin blocker crizanlizumab is an FDA-approved drug for SCD-associated pain crisis and priapism in the US [49], monitoring for liver parenchyma damage in patients on chronic administration should be considered for SCD patients.

Among all the LSEC markers examined, VCAM1 showed the strongest upregulation in SCD mouse livers [32]. VCAM1 expression in SCD patients positively correlated with increased risk of vaso-occlusive pain crisis and stroke [46]. Sterile inflammation and endothelial damage were also seen in SCD patients with VCAM1 activation [46]. Treatment with hydroxyurea caused a reduction in VCAM1 expression, hinting at a direct link between SCD-induced liver damage and LSEC activation [101].

ICAM1 is another endothelial marker, which exhibits enhanced expression and positively correlates with exacerbated vaso-occlusive pain crisis in SCD patients [102]. Previous research has demonstrated an association of ICAM1 with lymphocyte function-associated antigen 1 (LFA-1) and macrophage 1 antigen receptor (Mac1; CD11b/CD18) in models of oxidative stress and sterile inflammation in SCD [21]. Due to its diverse role in SCD pathophysiology, ICAM1 is considered a potential therapeutic target in SCD.

Among the other markers of ECs, members of the nitric oxide synthase (NOS) pathway, specifically iNOS and eNOS, are widely studied in SCD [34]. Enhanced expression of iNOS and eNOS was linked to vaso-occlusive pain crisis, sterile inflammation, and kidney damage. Both these endothelial markers are currently being investigated for their potential application as a therapeutic option for SCD. *Table 1 lists the endothelial cell-specific markers seen in LSECs of SCD mice.*

### 5.3. Altered Functional Properties of LSECs in SCD

A distinct characteristic of LSECs is the presence of scavenger receptors, which facilitate the removal of biomolecules [89]. These receptors exhibit high endocytic activity, which is important to regulate the hemostatic function of the liver. Among the most abundant scavenger receptors, stabilin 1, 2, and mannose receptors are worth mentioning [92]. These scavenger receptors facilitate immune surveillance, lipid metabolism, waste clearance, and endocytosis of micromolecules. Compared to littermate controls, SCD mice exhibit enhanced expression of scavenger receptors (stabilin 1 and 2) at baseline, which then gets significantly reduced upon oxyHb challenge (Figure 2C and preliminary data). Similar challenges with hemin, or LPS, which mimics the hemolytic insult or sterile inflammation seen in SCD patients, result in several-fold reductions in these LSEC-specific scavenger receptors (preliminary data) in SCD mice as compared to baseline. Many of these scavenger receptors participate in the clearance of phosphatidylserine-exposed cells, including aged RBCs [103]. Therefore, their increased expression at baseline could be a feedback mechanism to combat cell-free Hb-induced tissue damage, whereas loss of these receptors upon acute and chronic challenges could be an additional factor promoting the accumulation of aged RBCs and causing ischemia–reperfusion and inflammation in SCD. Changes in scavenger receptor dynamics can impact the outcome of liver-directed genetic manipulations in SCD and should be considered as a regulatory factor in future research. Additionally, a few components of the coagulation cascade, including FVIII [68], VWF [104], and TF [33], are cleared with the help of the scavenger receptors present in the LSECs. Thus, the prothrombotic phenotype seen in SCD could be associated with impaired/delayed clearance of these procoagulants due to loss of these scavenger receptors from the LSECs. Whereas scavenger receptors have not been connected to SCD pathophysiology, they are highly studied in disease models, including atherosclerosis, models of fibrosis, and metabolic disorders [92]. Targeting these scavenger receptors to stop the progression of liver dysfunction is considered a therapeutic strategy for these diseases. Thus, future work can be useful in understanding their role as potential biomolecules or therapeutic modulators in SCD.

Altered hemostasis is another common component of the pathophysiology of SCD [43]. LSECs play an indirect role in controlling hemostasis and venous thromboembolism (VTE) by serving as the primary source for the synthesis of several components involved in the coagulation cascade. VWF and FVIII are primarily synthesized by LSECs, and endothelial dysfunction in SCD correlates with elevated secretion of VWF and FVIII [65,105]. Recently, we (preliminary data) and others [106] have demonstrated that both acute and chronic challenges caused a significant increase in VWF expression in the LSECs of SCD, which correlated with exacerbated vaso-occlusive pain crises [104]. Among other coagulation markers, increased levels of D-dimers [42], TF [33], prothrombin fragment 1.2 [107], and thrombin-antithrombin complexes [108] are associated with endothelial damage in SCD. Impaired hemostasis (due to misexpression of these factors) has been connected to increased risk of vaso-occlusive pain crisis, stroke, and VTE [108]; thus, knowing the physiological involvement of LSECs in these processes may have potential therapeutic benefits in SCD.

### 5.4. LSECs Regulate Hb Clearance in SCD

**Figure 3:**
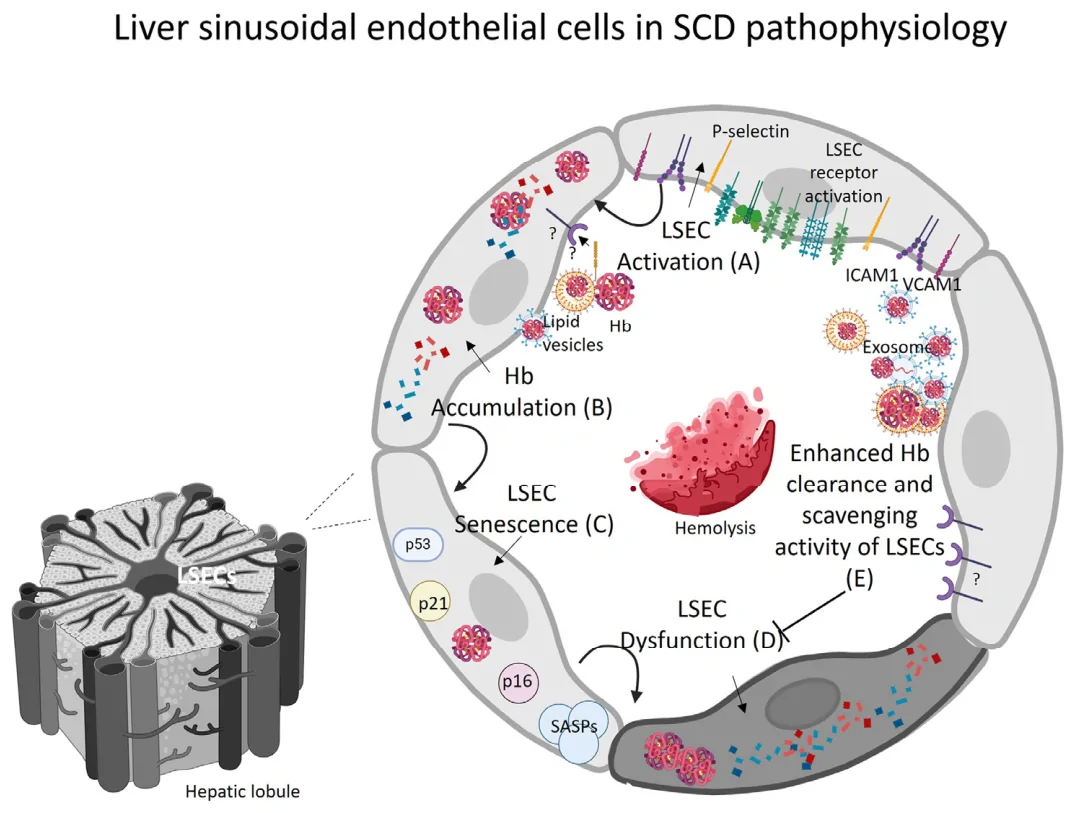
Liver sinusoidal endothelial cells in SCD pathophysiology. Schematic illustrating the function of LSECs in SCD pathophysiology. Hemolysis and accumulation of cell-free Hb facilitate the activation of LSECs (**A**), which express VCAM-1, ICAM-1, P-selectin, and VWF on their surface. These LSECs subsequently internalize Hb for clearance by micropinocytosis and potentially other undefined processes (**B**). Hb accumulation results in increased expression of senescence markers (P53, P21, and P16, etc.), causing LSEC senescence in SCD mice. (**C**). LSEC senescence shows a positive correlation with liver fibrosis (**D**). Accelerated Hb clearance or blocking of LSEC senescence can be developed as potential therapeutic strategies for SCD-associated liver damage (**E**). Image references: [53,93,95,96,114,118]. Created in BioRender. Pradhan, T. (2026) https://BioRender.com/n5nrogi.

Hemolysis plays a predominant role in the pathophysiology of SCD, causing cell-free Hb accumulation in different organs, primarily in the spleen and liver [2]. The adaptor protein haptoglobin binds to cell-free Hb and brings it to the liver and spleen for clearance [109,110,111,112]. Previous work has identified the presence of a specific Hb receptor, CD163 [113], which is abundantly present in circulating monocytes and macrophages, as well as in hepatic Kupffer cells. Ingested Hb metabolizes to form heme, which, through heme oxygenase 1 (HO-1) activity, further breaks down into iron, ferritin, carbon monoxide, and bilirubin/biliverdin. Persistent hemolysis eventually leads to reduced splenic activity and subsequent loss of haptoglobin, which promotes continuous accumulation of Hb in the liver [29]. Hb metabolites, heme, and iron also accumulate in the liver (Figure 3E), further aggravating the damage. Heme binds to adaptor protein hemopexin and gets cleared by the hepatic Kupffer cells [114]. Recently, it was shown that hepatic Kupffer cells undergo activation to a proinflammatory phenotype, which potentially can cause a loss of their phagocytic function [115,116]. When examined, we found accumulation of Hb in SCD mice and in patients’ liver samples at baseline, suggestive of their impaired/delayed clearance in SCD [53].

Subsequently, we [53] and others [84] have demonstrated that, along with hepatic Kupffer cells, LSECs also contribute to Hb clearance at baseline as well as in a preclinical mouse model (Figure 3) of SCD [53]. Both in vivo [53] and in vitro [117] analyses exhibited a strong co-localization of Hb with LSEC-specific markers. Zurawska et al. demonstrated that LSECs have a similar Hb clearance capacity to that of Kupffer cells in WT mice [84]. They further demonstrated micropinocytosis as the predominant route of LSEC-mediated Hb clearance [84]. Using the preclinical SCD Townes mouse model, we demonstrated accumulation of HbA and HbS in LSECs, which was depleted by nystatin- and latrunculin A-treated LSECs [53]. To further understand the mechanism of LSEC-specific Hb uptake, we utilized a pharmacological screen using small-molecule inhibitors of endocytosis in primary LSECs [117]. Our data showed that LSEC-specific Hb accumulation is a rather slow process. Mechanistically, we demonstrated a receptor-independent route as a predominant mode of LSEC-mediated Hb clearance by LSECs. We also showed that blocking clathrin-dependent endocytosis, lysosomal degradation, and lipid receptors can significantly inhibit Hb accumulation, indicating the possibility of alternate routes of Hb clearance by the LSECs [53,117].

LSECs are chiefly responsible for scavenging biomolecules as well as acting as a semipermeable barrier [91]. There were no previous reports of Hb clearance attributed to LSECs. Our data [53,117], along with that of others [84], suggest that under the pathophysiological conditions of SCD (e.g., hemolysis, hypoxia, and vaso-occlusion), in which Kupffer cell-mediated phagocytosis is depleted over time due to continuous accumulation of cell-free Hb, LSEC-mediated Hb clearance emerges as an alternate/parallel route to combat tissue damage [53]. Interestingly, we found that lung ECs can also promote Hb clearance, further supporting our hypothesis of a disease context-dependent role of ECs [53]. Subsequently, others have shown that compared to any other vascular endothelial cell, LSECs have the strongest capacity to internalize Hb through the process of micropinocytosis [84]. Future work will evaluate the pathophysiological significance and biomolecules involved in LSEC-mediated Hb clearance. The potential crosstalk of other liver cell types in promoting Hb clearance in SCD should also be evaluated in the future.

### 5.5. LSECs Undergo Senescence in SCD

Remarkably, one of the downstream consequences of LSEC-Hb accumulation was LSEC senescence, as seen by the increased expression of senescence markers, including P16, P21, and P53 (Figure 2D), and exacerbated release of SASPs (including P19INK4d, P16INK4a, MMP8, MMP3, MMP12, and Serpin AE1, etc.) from the LSECs of SCD mice [53]. Aggravated senescence was associated with liver damage in SCD [53,95]. Senescence-associated organ dysfunction is a common phenotype in many hematological disorders due to the accumulation of cell-free Hb, heme, and iron [118]. In vitro and in vivo studies confirmed that cell-free Hb accumulation is the major cause of LSEC senescence in SCD [53]. SCD mice were found to be more susceptible to oxidative stress and LSEC senescence compared to their littermate controls following hepatic Hb administration [53]. This could be either due to the absence of specific scavenger receptors in LSECs, resulting in inefficient or delayed Hb clearance, or increased polymerization of Hb inside LSECs, promoting oxidative stress and cellular damage. As SCD is known as a disease of accelerated aging [119] caused by premature hemolysis of sickle RBCs, LSEC senescence may be one of the downstream events of RBC hemolysis, further contributing to the accelerated aging process in SCD. Although limited information is available on the actual effect of LSEC senescence in SCD patients, previous research has shown a positive correlation of LSEC senescence with liver fibrosis, hepatocellular carcinoma, and impaired liver regeneration [120]. Thus, the use of senolytics as a preventative strategy to prevent LSEC dysfunction and liver damage in SCD may have therapeutic implications. It would be useful to examine the presence of senescence in other liver cell types, which are primarily involved in Hb clearance (Kupffer cells). Future research should focus on understanding the sequence of events leading to senescence in LSECs and ECs of other origins to improve our understanding of the molecular mechanisms of chronic organ dysfunction in SCD.

Despite the scarcity of available data on LSEC senescence in SCD patients, there are a few reports of positive correlation of senescence markers in various SCD contexts. Adolescent and young SCD patients are reported to exhibit an increased expression of senescence marker P16INK4a in their T lymphocytes, which positively correlates with exacerbated inflammation, tissue damage, and psychological decline [121]. Among other known senescent markers, the TP53 mutation in SCD patients may elevate the risk for myeloid malignancy [122]. The mutation may also impede the efficacy of gene therapy in these patients [123]. The P21 mutation was found in SCD patients with renal cell carcinoma [124]. Altogether, senescence might have a broader role in loss of SCD organ function, multiorgan failure, and overall mortality. Future studies will elucidate unknown functions of senescence in SCD.

## 6. Modulation of LSECs as a Potential Treatment Option for SCD-Associated Organ Damage

SCD patients experience both acute and chronic liver problems; however, a liver-directed approach mostly focuses on iron elimination post-blood transfusion. Recent data from our lab and that of others suggest that LSECs play a specific role in SCD pathophysiology, albeit different from other EC types. These findings highlight the potential usefulness of LSECs in liver-directed therapeutic approaches to SCD. While prior research did not indicate a role for LSECs or other ECs in hemoglobin clearance, our recent findings propose the potential for an alternative mechanism of EC-mediated hemoglobin clearance. Identifying new regulators of LSEC-mediated Hb clearance may have therapeutic implications for SCD and other hemolytic disorders such as thalassemia, sepsis, and paroxysmal nocturnal hemoglobinuria. Similarly, it would be therapeutically advantageous to investigate the direct and indirect roles of LSECs in the clearance of heme and iron to potentially identify new biomarkers and regulators of these processes. The observed reduction in LSEC-specific scavenging receptors (stabilin 1 and 2) following cell-free Hb accumulation suggests a potential mechanism of LSEC dysfunction via aberrant endocytosis. Forced expression of these scavenger receptors may confer protective benefits against liver and other organ damage caused by Hb, heme, and iron. Future study examining the viability of employing these receptors as potential modulators of hemolysis-induced chronic organ damage might be beneficial. Given that LSECs are the primary source of synthesis of several procoagulants associated with heightened vaso-occlusive crises and VTE in SCD, the impact of LSEC dysfunction on the control of SCD-related coagulation impairment warrants comprehensive investigation in future studies. Among other methods of LSEC manipulations, its specific location (juxtaposed with hepatic Kupffer cells and hepatocytes) could be beneficial for hepatic drug delivery purposes. Preclinical mouse models with LSEC manipulation (such as LSEC-specific knockdown of scavenging receptors) may aid in understanding the role of LSECs in SCD pathophysiology. CECs are now recognized biomarkers of SCD-induced vaso-occlusive pain crisis and multi-organ damage; LSEC-specific markers may also emerge in the future as noninvasive biomarkers of SCD-induced organ damage. Future research should also examine the involvement of exosomes released from LSECs in the amelioration of liver fibrosis and the clearance of Hb, iron, or other microparticles associated with SCD.

## 7. Conclusions

While contemporary treatment modalities are expanding the lifespan of SCD patients, there are reports of increased hospitalization due to chronic organ damage, including liver damage. In this review, we have discussed the novel roles of LSECs in promoting SCD pathophysiology with potential novel therapeutic treatment options. Although LSECs constitute a minor fraction of total liver cells, they hold particular importance in the liver due to their strategic location as the first line of defense, forming a semi-permeable barrier with blood and liver, as well as their remarkable scavenging function. Recent studies have elucidated numerous distinct alterations in LSECs due to hemolysis and cell-free Hb accumulation, as discussed in this review. Understanding the molecular mechanism and signaling molecules involved can open many new therapeutic modalities for SCD and other hemolytic disorders. Ongoing studies examining the effect of LSEC dysfunction in causing multiorgan damage and impaired hepatic regeneration may reveal the previously unknown role of these cells in SCD and other hemolytic diseases. Genetic manipulations, as well as lineage tracing studies in the future, can be particularly useful to comprehend the therapeutic potential of these cells in hemolytic disease contexts. There exists a constraint on the translational interpretation of the preclinical studies discussed here, owing to the difficulties associated with investigating LSECs in SCD patients. Future ex vivo and organoid research may provide valuable insights into the role of these cells in sickle cell disease pathophysiology and other similar hemolytic disorders.

## Data Availability

No new data was analyzed during this study.
